# Supplementary material for: A Likelihood Approach for Real-Time Calibration of Stochastic Compartmental Epidemic Models
Source: PLoS Comput Biol. 2017 Jan 17;13(1):e1005257. doi: 10.1371/journal.pcbi.1005257 (PMC5240920; doi:10.1371/journal.pcbi.1005257)
Supplement: S1 File — (TAR.GZ) [file pcbi.1005257.s014.tar.gz › HSPH_Online-SI-Revision/IPoi09/n100-extreme/n100-extreme_BENCH2_table0.pdf]

1	newly 3 weeks spec	7253.	7212.66	{4444., 10457.}	24.4204	0.106832	newly 3 weeks cumul	27162.	26288.3	{18510., 33974.}	17.0066	0.0794822	newly 5 weeks spec	4336.	4362.89	{2335., 8009.}	37.0741	0.15375	newly 5 weeks cumul	37280.	36339.4	{24277., 48714.}	19.8584	0.0898033	newly 54 weeks cumul	48774.	47890.3	{29849., 75493.}	25.2208	0.11094
2	newly 3 weeks spec	5965.	3768.23	{2085., 6516.}	47.4437	0.283919	newly 3 weeks cumul	20994.	13957.7	{8450., 23409.}	40.5746	0.236902	newly 5 weeks spec	3796.	2371.75	{1221., 4091.}	53.1219	0.318263	newly 5 weeks cumul	29744.	19342.4	{11285., 32661.}	43.9764	0.259077	newly 50 weeks cumul	42279.	27105.1	{15043., 46010.}	47.7754	0.282788
3	newly 3 weeks spec	6513.	4929.11	{2527., 7571.}	42.0966	0.229283	newly 3 weeks cumul	27002.	20382.9	{11810., 31030.}	34.6271	0.191289	newly 5 weeks spec	3181.	2422.57	{1124., 3822.}	48.4454	0.260246	newly 5 weeks cumul	34839.	26312.5	{14625., 40393.}	37.3735	0.205336	newly 55 weeks cumul	41209.	31294.	{16824., 47963.}	39.3318	0.214337
4	newly 3 weeks spec	6774.	6959.37	{4172., 10875.}	33.4883	0.139193	newly 3 weeks cumul	33063.	32169.1	{22069., 45433.}	22.6188	0.103114	newly 5 weeks spec	2428.	2613.48	{1378., 4119.}	40.9444	0.155735	newly 5 weeks cumul	39680.	39141.9	{25767., 57933.}	25.3545	0.112854	newly 59 weeks cumul	42886.	42812.2	{27683., 63473.}	26.808	0.117096
5	newly 3 weeks spec	8431.	6720.07	{3743., 10358.}	35.3494	0.187947	newly 3 weeks cumul	31964.	26150.2	{17073., 37303.}	25.0319	0.132144	newly 5 weeks spec	4440.	3507.1	{1659., 5541.}	45.0321	0.236442	newly 5 weeks cumul	42439.	34586.4	{21284., 51109.}	29.034	0.152926	newly 56 weeks cumul	51326.	41990.5	{24614., 62501.}	32.4466	0.168864
6	newly 3 weeks spec	7676.	4362.89	{2335., 8009.}	50.805	0.319448	newly 3 weeks cumul	28666.	17263.8	{10046., 28689.}	43.4738	0.264467	newly 5 weeks spec	4357.	2393.74	{1115., 4287.}	57.3671	0.367439	newly 5 weeks cumul	38952.	22907.3	{12877., 40484.}	46.7708	0.288258	newly 54 weeks cumul	50195.	28814.7	{15576., 51228.}	49.8826	0.311199
7	newly 3 weeks spec	7326.	7720.52	{5412., 10233.}	20.0347	0.0861656	newly 3 weeks cumul	27951.	25545.6	{19021., 31295.}	14.4513	0.0755085	newly 5 weeks spec	3953.	5584.92	{3210., 9662.}	49.6042	0.159266	newly 5 weeks cumul	37123.	37783.7	{27807., 47847.}	15.7357	0.0697184	newly 52 weeks cumul	46099.	57482.3	{37465., 83764.}	32.7715	0.118049
8	newly 3 weeks spec	7809.	5688.23	{3225., 9960.}	38.0099	0.209358	newly 3 weeks cumul	28877.	21705.1	{13838., 30976.}	29.6691	0.160784	newly 5 weeks spec	4404.	3249.76	{1575., 5849.}	47.2719	0.252871	newly 5 weeks cumul	39237.	29317.7	{17925., 44633.}	32.8414	0.17865	newly 54 weeks cumul	50550.	37618.9	{21745., 63980.}	37.3186	0.201578
9	newly 3 weeks spec	8491.	8202.83	{5074., 11358.}	23.8354	0.111298	newly 3 weeks cumul	32010.	28169.8	{19693., 34398.}	16.6016	0.0835498	newly 5 weeks spec	4649.	5268.22	{2729., 9465.}	42.1772	0.162639	newly 5 weeks cumul	43099.	40148.3	{27419., 51605.}	18.4119	0.0888626	newly 54 weeks cumul	54004.	54187.1	{33743., 79890.}	25.9276	0.114915
10	newly 3 weeks spec	3970.	6358.2	{3607., 11159.}	65.4557	0.18495	newly 3 weeks cumul	22883.	28662.8	{18780., 45181.}	34.6418	0.121537	newly 5 weeks spec	1101.	2511.43	{1273., 4569.}	128.917	0.299412	newly 5 weeks cumul	26005.	35232.	{22301., 56843.}	42.5435	0.138896	newly 58 weeks cumul	27241.	39033.	{24193., 63798.}	49.1628	0.153999
11	newly 3 weeks spec	6532.	5449.66	{3048., 9469.}	33.6535	0.172139	newly 3 weeks cumul	23257.	20132.2	{12750., 28717.}	24.6888	0.125125	newly 5 weeks spec	3974.	3296.2	{1631., 5855.}	42.6914	0.213426	newly 5 weeks cumul	32341.	27702.4	{16579., 45738.}	28.9377	0.146174	newly 52 weeks cumul	43101.	37024.9	{20951., 62619.}	33.4798	0.166017
12	newly 3 weeks spec	8024.	7970.84	{5501., 10428.}	19.922	0.0896697	newly 3 weeks cumul	31244.	27014.7	{20007., 32569.}	15.2601	0.0801088	newly 5 weeks spec	4154.	5449.66	{3048., 9469.}	43.4134	0.146056	newly 5 weeks cumul	41376.	39176.	{28628., 49343.}	15.6098	0.0737722	newly 53 weeks cumul	50863.	56068.8	{36957., 81733.}	25.0753	0.100658
13	newly 3 weeks spec	7496.	5572.89	{3524., 8114.}	31.8828	0.176382	newly 3 weeks cumul	24982.	19616.6	{13781., 26192.}	24.1051	0.129347	newly 5 weeks spec	4998.	3768.23	{2085., 6516.}	41.9696	0.225809	newly 5 weeks cumul	36270.	28001.4	{18668., 39225.}	27.6756	0.150184	newly 51 weeks cumul	54152.	41148.8	{25261., 65972.}	35.0631	0.1892
14	newly 3 weeks spec	5024.	9982.21	{7322., 12729.}	99.0212	0.288872	newly 3 weeks cumul	30056.	32560.7	{25766., 37667.}	18.8653	0.0849707	newly 5 weeks spec	1153.	6696.69	{3867., 11179.}	480.806	0.732592	newly 5 weeks cumul	33840.	47635.9	{37932., 55558.}	41.6598	0.147004	newly 56 weeks cumul	34818.	66071.5	{47494., 87424.}	89.7623	0.26786
15	newly 3 weeks spec	5483.	7298.18	{4564., 11659.}	40.7874	0.136487	newly 3 weeks cumul	26035.	28784.5	{20177., 39484.}	21.0663	0.0843319	newly 5 weeks spec	1932.	3699.21	{2103., 6155.}	92.4706	0.239672	newly 5 weeks cumul	31301.	37761.4	{25306., 55886.}	29.1148	0.107307	newly 56 weeks cumul	33894.	45363.4	{29205., 68719.}	39.7129	0.134114
16	newly 3 weeks spec	6616.	5277.76	{3127., 8770.}	37.606	0.192196	newly 3 weeks cumul	30832.	22162.9	{14179., 34962.}	33.8507	0.18749	newly 5 weeks spec	2584.	2525.82	{1352., 4296.}	39.3432	0.16476	newly 5 weeks cumul	37486.	28387.9	{17634., 45413.}	34.4886	0.184478	newly 56 weeks cumul	41249.	33464.	{20181., 53672.}	34.2913	0.173983
17	newly 3 weeks spec	6952.	6973.77	{4541., 9848.}	23.1152	0.102363	newly 3 weeks cumul	24739.	23672.	{16822., 30167.}	16.5535	0.0791442	newly 5 weeks spec	4470.	4878.31	{2610., 8636.}	36.4614	0.144164	newly 5 weeks cumul	34818.	34479.2	{23538., 46041.}	19.2175	0.0863622	newly 51 weeks cumul	48456.	51559.9	{31343., 78378.}	27.6505	0.113583
18	newly 3 weeks spec	7355.	7976.1	{5517., 10469.}	21.0412	0.0893156	newly 3 weeks cumul	26994.	25764.4	{19927., 30611.}	13.7076	0.0725917	newly 5 weeks spec	4020.	5928.95	{3499., 9374.}	52.6404	0.167256	newly 5 weeks cumul	36463.	38667.2	{28675., 47645.}	16.6767	0.0716607	newly 52 weeks cumul	45100.	60626.1	{39949., 84814.}	38.6419	0.133712
19	newly 3 weeks spec	8310.	8202.83	{5074., 11358.}	24.1368	0.110362	newly 3 weeks cumul	31919.	28169.8	{19693., 34398.}	16.5634	0.0831789	newly 5 weeks spec	4512.	5268.22	{2729., 9465.}	43.2399	0.16171	newly 5 weeks cumul	42585.	40148.3	{27419., 51605.}	18.4732	0.0881678	newly 54 weeks cumul	52753.	54187.1	{33743., 79890.}	26.3053	0.113897
20	newly 3 weeks spec	6547.	9455.74	{5978., 15377.}	49.8772	0.157363	newly 3 weeks cumul	38691.	37942.9	{28639., 48944.}	17.2053	0.0775514	newly 5 weeks spec	1542.	4057.72	{2110., 7093.}	163.147	0.363985	newly 5 weeks cumul	43499.	48358.8	{34569., 67178.}	23.6477	0.093771	newly 59 weeks cumul	44967.	54671.3	{37734., 79946.}	29.6808	0.107449
21	newly 3 weeks spec	7119.	8410.21	{5580., 13525.}	30.8441	0.113836	newly 3 weeks cumul	29356.	30981.8	{23749., 37734.}	16.4422	0.0689329	newly 5 weeks spec	3172.	4633.61	{2643., 8455.}	54.7126	0.159483	newly 5 weeks cumul	37313.	41990.1	{30320., 57489.}	22.9282	0.0899278	newly 56 weeks cumul	42719.	52166.7	{35851., 79394.}	30.5326	0.109497
22	newly 3 weeks spec	6759.	7825.4	{5116., 12322.}	31.1429	0.116271	newly 3 weeks cumul	29716.	31250.3	{23027., 41912.}	19.2169	0.0810656	newly 5 weeks spec	2827.	3791.1	{2115., 6056.}	47.7903	0.148628	newly 5 weeks cumul	37047.	40599.9	{28446., 57398.}	23.4997	0.0944713	newly 57 weeks cumul	41620.	47792.1	{32351., 69953.}	27.7454	0.105291
23	newly 3 weeks spec	6270.	5572.89	{3524., 8114.}	26.8737	0.132271	newly 3 weeks cumul	22584.	19616.6	{13781., 26192.}	20.6921	0.104798	newly 5 weeks spec	3912.	3768.23	{2085., 6516.}	37.3118	0.164108	newly 5 weeks cumul	31596.	28001.4	{18668., 39225.}	23.8318	0.118313	newly 50 weeks cumul	43028.	41148.8	{25261., 65972.}	30.1137	0.135907
24	newly 3 weeks spec	7567.	6289.55	{4350., 8422.}	22.1072	0.114805	newly 3 weeks cumul	27322.	21508.9	{14772., 27367.}	21.5846	0.11689	newly 5 weeks spec	4888.	4496.25	{2769., 6985.}	29.7596	0.140599	newly 5 weeks cumul	38440.	31380.8	{23233., 40856.}	20.9327	0.10922	newly 50 weeks cumul	52801.	48462.5	{32593., 69031.}	24.9013	0.117867
25	newly 3 weeks spec	6782.	7970.84	{5501., 10428.}	26.792	0.103044	newly 3 weeks cumul	30207.	27014.7	{20007., 32569.}	14.2984	0.0737764	newly 5 weeks spec	2743.	5449.66	{3048., 9469.}	99.4161	0.267106	newly 5 weeks cumul	37399.	39176.	{28628., 49343.}	17.3553	0.0742402	newly 53 weeks cumul	41988.	56068.8	{36957., 81733.}	38.9857	0.135294
26	newly 3 weeks spec	7069.	5928.95	{3499., 9374.}	29.9068	0.153603	newly 3 weeks cumul	23874.	20878.8	{13723., 28685.}	22.3397	0.111964	newly 5 weeks spec	4701.	3952.47	{1992., 7191.}	40.7727	0.205158	newly 5 weeks cumul	34401.	29709.6	{18471., 42859.}	25.8918	0.130457	newly 51 weeks cumul	50936.	42837.8	{24342., 69397.}	32.9557	0.167093
27	newly 3 weeks spec	6335.	4212.81	{2194., 7453.}	43.926	0.258037	newly 3 weeks cumul	22631.	16216.7	{9438., 25578.}	36.3281	0.204937	newly 5 weeks spec	3937.	2467.16	{1219., 4488.}	50.8607	0.307108	newly 5 weeks cumul	31837.	21930.5	{12309., 37708.}	40.1263	0.230991	newly 52 weeks cumul	45079.	28874.2	{15572., 50176.}	45.1196	0.269567
28	newly 3 weeks spec	7243.	4364.77	{2349., 6239.}	49.4408	0.300087	newly 3 weeks cumul	27640.	19380.8	{11289., 27835.}	38.1261	0.214587	newly 5 weeks spec	4186.	1908.73	{928., 2812.}	60.2031	0.421001	newly 5 weeks cumul	37414.	24201.7	{13760., 34906.}	43.0335	0.252031	newly 56 weeks cumul	48001.	27616.7	{15548., 39874.}	48.4571	0.300576
29	newly 3 weeks spec	6769.	7058.79	{5121., 9199.}	19.9078	0.0870178	newly 3 weeks cumul	23489.	23613.2	{18680., 28273.}	14.2609	0.0687484	newly 5 weeks spec	4398.	5077.9	{3294., 7473.}	30.8799	0.116821	newly 5 weeks cumul	33395.	34751.3	{26267., 43394.}	16.7031	0.0727415	newly 50 weeks cumul	46315.	54038.6	{37690., 73214.}	26.5243	0.0998061
30	newly 3 weeks spec	8192.	4359.24	{2368., 7061.}	53.267	0.34481	newly 3 weeks cumul	34290.	21883.6	{13385., 33967.}	41.0916	0.244083	newly 5 weeks spec	3775.	1542.82	{819., 2281.}	64.0927	0.466267	newly 5 weeks cumul	43617.	26039.9	{15550., 40367.}	45.0025	0.275506	newly 59 weeks cumul	50418.	28167.4	{16634., 43626.}	48.3216	0.30444
31	newly 3 weeks spec	7625.	7869.65	{4691., 12699.}	32.0558	0.130264	newly 3 weeks cumul	37142.	36710.	{26116., 49323.}	19.1787	0.0858985	newly 5 weeks spec	2494.	2734.71	{1467., 4458.}	40.1557	0.147071	newly 5 weeks cumul	43977.	44163.8	{30235., 64888.}	22.1568	0.0963072	newly 60 weeks cumul	46875.	47622.3	{32131., 70220.}	23.4815	0.100109
32	newly 3 weeks spec	6240.	7825.4	{5116., 12322.}	35.9132	0.125411	newly 3 weeks cumul	27473.	31250.3	{23027., 41912.}	22.6756	0.0890052	newly 5 weeks spec	2353.	3791.1	{2115., 6056.}	66.5808	0.185369	newly 5 weeks cumul	33807.	40599.9	{28446., 57398.}	28.5445	0.106122	newly 57 weeks cumul	37286.	47792.1	{32351., 69953.}	35.1104	0.122421
33	newly 3 weeks spec	8467.	6696.69	{3867., 11179.}	33.7155	0.179266	newly 3 weeks cumul	28949.	25057.4	{16616., 34404.}	21.6662	0.110073	newly 5 weeks spec	5209.	3825.29	{1984., 6767.}	45.8933	0.251087	newly 5 weeks cumul	40750.	34055.2	{21372., 51726.}	26.9736	0.139046	newly 55 weeks cumul	56254.	43493.	{25733., 70048.}	33.7565	0.18097
34	newly 3 weeks spec	6053.	6074.92	{3914., 8501.}	23.5437	0.103612	newly 3 weeks cumul	19976.	21151.2	{15472., 27019.}	19.2292	0.0826674	newly 5 weeks spec	4286.	4160.33	{2562., 6254.}	30.6962	0.136531	newly 5 weeks cumul	29425.	30404.6	{21103., 40870.}	21.3534	0.0914389	newly 50 weeks cumul	43351.	45585.2	{29923., 64214.}	26.9688	0.111359
35	newly 3 weeks spec	8823.	6696.69	{3867., 11179.}	34.9583	0.190805	newly 3 weeks cumul	31843.	25057.4	{16616., 34404.}	24.8512	0.133504	newly 5 weeks spec	5272.	3825.29	{1984., 6767.}	46.1929	0.254792	newly 5 weeks cumul	44053.	34055.2	{21372., 51726.}	29.4328	0.159247	newly 55 weeks cumul	57367.	43493.	{25733., 70048.}	34.4138	0.186725
36	newly 3 weeks spec	6496.	3810.	{2008., 7154.}	50.267	0.315147	newly 3 weeks cumul	23327.	15044.5	{8533., 26935.}	43.3755	0.258471	newly 5 weeks spec	4189.	2120.26	{1032., 3850.}	56.9959	0.38723	newly 5 weeks cumul	32804.	20021.8	{10897., 35920.}	46.7155	0.286714	newly 53 weeks cumul	44915.	25585.4	{13583., 46206.}	50.2502	0.319751
37	newly 3 weeks spec	6929.	8541.05	{5202., 14396.}	37.0311	0.131925	newly 3 weeks cumul	31730.	34610.4	{24780., 45365.}	21.3406	0.0874022	newly 5 weeks spec	2540.	3790.05	{2034., 7133.}	58.8596	0.173931	newly 5 weeks cumul	38553.	44227.2	{30190., 65091.}	26.7569	0.103743	newly 58 weeks cumul	42395.	50619.5	{33660., 77760.}	30.6733	0.113818
38	newly 3 weeks spec	5419.	8541.05	{5202., 14396.}	60.4742	0.183367	newly 3 weeks cumul	32641.	34610.4	{24780., 45365.}	20.154	0.0847988	newly 5 weeks spec	1229.	3790.05	{2034., 7133.}	208.385	0.442053	newly 5 weeks cumul	36650.	44227.2	{30190., 65091.}	29.7758	0.110651	newly 58 weeks cumul	37768.	50619.5	{33660., 77760.}	39.1903	0.133414
39	newly 3 weeks spec	5970.	7720.52	{5412., 10233.}	34.7107	0.126289	newly 3 weeks cumul	21972.	25545.6	{19021., 31295.}	24.1932	0.0991639	newly 5 weeks spec	3152.	5584.92	{3210., 9662.}	78.6981	0.223696	newly 5 weeks cumul	29579.	37783.7	{27807., 47847.}	31.368	0.115495	newly 52 weeks cumul	36448.	57482.3	{37465., 83764.}	58.6009	0.184918
40	newly 3 weeks spec	6214.	5268.22	{2729., 9465.}	38.4902	0.195664	newly 3 weeks cumul	22734.	20181.3	{11566., 33204.}	29.0362	0.145125	newly 5 weeks spec	3630.	2924.48	{1320., 5296.}	46.5468	0.243992	newly 5 weeks cumul	31216.	27066.8	{14766., 45534.}	32.9448	0.166154	newly 53 weeks cumul	41146.	34220.1	{17890., 58730.}	37.1528	0.191725
41	newly 3 weeks spec	5376.	7212.66	{4444., 10457.}	40.4279	0.137364	newly 3 weeks cumul	21206.	26288.3	{18510., 33974.}	30.0892	0.112936	newly 5 weeks spec	2830.	4362.89	{2335., 8009.}	61.0698	0.174878	newly 5 weeks cumul	27896.	36339.4	{24277., 48714.}	35.3075	0.12528	newly 54 weeks cumul	33607.	47890.3	{29849., 75493.}	47.0243	0.153215
42	newly 3 weeks spec	4668.	5826.8	{3267., 10511.}	42.525	0.146473	newly 3 weeks cumul	24350.	26037.5	{16356., 42444.}	27.927	0.114421	newly 5 weeks spec	1451.	2413.08	{1278., 4656.}	71.6691	0.19568	newly 5 weeks cumul	28470.	32240.6	{19729., 54233.}	32.0676	0.123235	newly 58 weeks cumul	30111.	36219.8	{21838., 62059.}	36.2545	0.131093
43	newly 3 weeks spec	8069.	7502.05	{5522., 9707.}	17.8493	0.0868669	newly 3 weeks cumul	29202.	24586.6	{18086., 29583.}	16.304	0.0908983	newly 5 weeks spec	4750.	5572.89	{3524., 8114.}	33.0954	0.125204	newly 5 weeks cumul	40208.	36701.2	{28219., 44955.}	15.1002	0.0742145	newly 51 weeks cumul	53458.	58233.4	{40612., 81952.}	24.0785	0.0976251
44	newly 3 weeks spec	6354.	3790.05	{2034., 7133.}	48.0877	0.298069	newly 3 weeks cumul	27932.	18157.9	{10564., 32186.}	41.9637	0.247667	newly 5 weeks spec	2650.	1514.08	{798., 2846.}	51.4723	0.325955	newly 5 weeks cumul	34711.	22085.1	{12657., 39837.}	43.5994	0.260241	newly 57 weeks cumul	39102.	24550.1	{13951., 44668.}	44.584	0.268028
45	newly 3 weeks spec	4797.	7298.18	{4564., 11659.}	55.1995	0.171397	newly 3 weeks cumul	26104.	28784.5	{20177., 39484.}	20.9254	0.0839611	newly 5 weeks spec	1420.	3699.21	{2103., 6155.}	160.508	0.368564	newly 5 weeks cumul	30202.	37761.4	{25306., 55886.}	32.053	0.115321	newly 56 weeks cumul	31840.	45363.4	{29205., 68719.}	46.0937	0.150193
46	newly 3 weeks spec	8356.	8733.67	{5501., 12891.}	26.7941	0.114071	newly 3 weeks cumul	33903.	31557.3	{22268., 39733.}	16.0021	0.0771805	newly 5 weeks spec	3539.	4929.11	{2527., 7571.}	53.5184	0.165643	newly 5 weeks cumul	42756.	43206.5	{30079., 57374.}	19.8033	0.0876436	newly 56 weeks cumul	48435.	54117.6	{35093., 75750.}	27.3042	0.10797
47	newly 3 weeks spec	6682.	8541.05	{5202., 14396.}	39.4591	0.136445	newly 3 weeks cumul	30081.	34610.4	{24780., 45365.}	24.1309	0.0942662	newly 5 weeks spec	2681.	3790.05	{2034., 7133.}	53.9229	0.165714	newly 5 weeks cumul	37114.	44227.2	{30190., 65091.}	28.9731	0.10877	newly 58 weeks cumul	40818.	50619.5	{33660., 77760.}	33.1555	0.119348
48	newly 3 weeks spec	8702.	8541.05	{5202., 14396.}	28.3843	0.125684	newly 3 weeks cumul	37680.	34610.4	{24780., 45365.}	17.2648	0.0835535	newly 5 weeks spec	3585.	3790.05	{2034., 7133.}	37.5691	0.150373	newly 5 weeks cumul	46811.	44227.2	{30190., 65091.}	20.8101	0.0972678	newly 58 weeks cumul	52174.	50619.5	{33660., 77760.}	23.0081	0.103955
49	newly 3 weeks spec	4127.	5671.29	{3186., 8377.}	50.1841	0.1535	newly 3 weeks cumul	27465.	30086.	{19261., 43463.}	29.7232	0.117398	newly 5 weeks spec	921.	1703.02	{910., 2415.}	87.0635	0.21919	newly 5 weeks cumul	30359.	34944.8	{21857., 50576.}	32.8522	0.122421	newly 60 weeks cumul	31206.	36902.5	{22891., 53318.}	34.5225	0.124925
50	newly 3 weeks spec	6476.	6653.21	{4242., 9757.}	25.4834	0.109405	newly 3 weeks cumul	23838.	23001.5	{16218., 30176.}	17.1917	0.0804174	newly 5 weeks spec	3992.	4544.94	{2427., 8122.}	40.1852	0.150075	newly 5 weeks cumul	32964.	33131.3	{22278., 44671.}	21.2347	0.0936978	newly 51 weeks cumul	44879.	48284.9	{29128., 76423.}	29.5003	0.118993
